# Analysis of polypeptide expression in benign and malignant human breast lesions: down-regulation of cytokeratins.

**DOI:** 10.1038/bjc.1996.600

**Published:** 1996-11

**Authors:** B. Franzén, S. Linder, A. A. Alaiya, E. Eriksson, K. Uruy, T. Hirano, K. Okuzawa, G. Auer

**Affiliations:** Unit of Cell and Molecular Analysis, Karolinska Institute and Hospital, Stockholm, Sweden.

## Abstract

**Images:**


British Journal of Cancer (1996) 73, 1632–1638
© 1996 Stockton Press All rights reserved 0007–0920/96 $12.00

### Analysis of polypeptide expression in benign and malignant human breast lesions: down-regulation of cytokeratins

B Franzén[1], S Linder[2], AA Alaiya[1], E Eriksson[1], K Uruy[3], T Hirano[3], K Okuzawa[3] and G Auer[1]

*[1]Unit of Cell and Molecular Analysis and [2]Radiumhemmets Research Laboratory, Department of Oncology and Pathology, Karolinska Institute and Hospital, S-171 76 Stockholm, Sweden; [3]Department of Surgery, Tokyo Medical College, Nishishinjuku, Shinjukuku, Tokyo, Japan.*

**Summary** Malignant progression of tumour cells is caused by the accumulation of genetic defects, which when combined will generate a large phenotypic diversity. Simultaneous quantitation of a large number of gene products in tumour cells is desirable, but difficult to achieve. We have here quantitated the levels of a number of abundant polypeptides in human breast carcinoma cells using two-dimensional gel electrophoresis (2-DE; PDQUEST). For this purpose, tumour cells were prepared from the tissue of 17 breast carcinomas. Fibroadenoma tissue was used as reference for benign cells. An increase of the spot density of the PCNA polypeptide was observed in rapidly proliferating tumour cells, confirming the validity of the procedures used. In the set of 24 polypeptide spots with known identity, decreases in cytokeratin and tropomyosin levels were observed. The levels of all cytokeratin forms resolved (CK7, CK8, CK15 and CK18) were significantly lower in carcinomas than in fibroadenomas. The levels of tropomyosin 2 and 3 were lower in carcinomas than in fibroadenomas. In contrast, the levels of some members of the stress protein family (pHSP60, HSP90 and calreticulin) were higher in carcinomas. Furthermore, changes in the expression of lactate dehydrogenase and GT-$\pi$, but not in nm23, were observed. We conclude that simultaneous analysis of multiple polypeptides in human carcinomas can be achieved by 2-DE and may be useful in prognostic studies, and that malignant progression of breast carcinomas results in the decreased expression of cytokeratin polypeptides. This phenomenon must be considered in studies where cytokeratins are used as markers to identify the epithelial cell compartment in breast carcinomas.

**Keywords:** two-dimensional gel electrophoresis; human breast tumour; cytokeratin

Breast cancer is both biologically and clinically a heterogeneous disease. Although presenting without evidence of disseminating cancer, a proportion of women will die rapidly in metastatic disease. In spite of enormous efforts in breast cancer research, three main problems remain: (1) to objectively and reliably select those premalignant lesions which, if untreated, will progress to invasive malignancy; (2) to objectively and reliably determine the aggressiveness of an individual tumour and (3) to analyse cellular properties which allow highly individualised tumour-specific treatment.

Two-dimensional gel electrophoresis (2-DE) is a technique which can be used to obtain qualitative and quantitative information on protein expression in cells. In a recent update of the two-dimensional protein database, 1082 proteins were reported to be identified by name (Celis *et al.*, 1995). Identification is aided by recent progress in microsequencing, including mass spectrophotometry. We have here explored 2-DE to characterise polypeptide profiles in human breast carcinomas. Major polypeptides in the gel profiles were identified (mostly cytoskeletal and stress-related proteins) and quantified. We describe alterations in the expression of some of these polypeptides. We show that the levels of several cytokeratin polypeptides are lower in carcinomas than in fibroadenomas. These results suggest that 2-DE can be a valuable future tool for the characterisation of gene expression in human malignant tumours.

### Materials and methods

### *Tumour tissue samples*

All samples, described in Table I, were obtained shortly after resection and processed essentially as described below (Franzén *et al.*, 1993). Cells were purified from non-necrotic tumour tissue within 40 min after resection, and all steps were performed on ice in the presence of protease inhibitors.

Each resected tumour was placed on ice, cut in the middle and one (or two) macroscopically representative and non-necrotic area was selected for extraction of cells. The surface of a freshly cut tumour was scraped with the dry blade of a sharp scalpel. As previously discussed, tumour cells are more loosely attached to the extracellular matrix than normal cells and will be preferentially extracted. Cells were collected in 1–2 ml of ice-cold medium (RPMI-1640) supplemented with 5% calf serum/protease inhibitors (0.2 mM phenylmethylsulphonyl fluoride and 0.83 mM benzamidine). Cell suspensions were first filtered using a 250 $\mu$m filter directly followed by a 100 $\mu$m nylon mesh to remove tissue fragments. Cell suspensions were then collected in new tubes and carefully underlaid with 1.0–1.5 ml of ice-cold Percoll phosphate-buffered saline (PBS) solution (54.7%, density 1.07 g ml$^{-1}$) and finally centrifuged for 10 min at 1000 ***g*** and 4°C. The interface cell layers were washed with PBS and pelleted. The wet weight of each cell pellet was recorded and cells were then stored at −80°C. The final preparation of cell pellets was performed according to Linder *et al.* (1979) and Garrels *et al.* (1979), with some modifications (Franzén *et al.*, 1993). Adjacent material was fixed in 4% buffered formalin and embedded in paraffin.

### *Characterisation of formalin-fixed specimens*

Malignant tumours were subdivided into two subgroups on the basis of proliferative index and DNA ploidy assessments. Proliferation index was determined by immunohistochemical staining using the MIB-1 antibody (Immunotech). The fraction of positive cells was scored and classified as 'low' (<20%), 'intermediate' (20–50%) and 'high' (>50%). Histopathology (using haematoxylin–eosin-stained sections) was performed by one experienced pathologist and classified according to WHO. The nuclear DNA content was assessed using image cytometric analysis of Feulgen-stained

Correspondence: B Franzén
Received 18 March 1996; revised 14 June 1996; accepted 2 July 1996

**Table I** Clinical data and characteristics of the samples analysed

| *Case No.* | *Histopathological Diagnosis* | *Patient age (years)* | *Size (mm)* | *Histopathological Differentiation*[a] | *Proliferation (MIB-1)*[b] | *Lymph nodes positive/total* |
|---|---|---|---|---|---|---|
| *Non-malignant lesions* | | | | | | |
| 141 | Normal | – | – | – | ND | – |
| 122 | Fibroadenoma | 21 | 20 | – | L | – |
| 124 | Fibroadenoma | 20 | 20 | – | IM | – |
| 127 | Hamartoma | 32 | 25 | – | L | – |
| 128 | Fibroadenoma | 32 | 7 | – | L | – |
| 139 | Fibroadenomatosis | 20 | 17 | – | IM | – |
| 140 | Fibroadenoma | 20 | 50 | – | ND | – |
| *DNA-diploid and low-proliferative invasive carcinomas* | | | | | | |
| 012 | | 78 | 22 | M | L | 0/4 |
| 071 | | 66 | 14 | P | L | 0/3 |
| 088 | | 78 | 27 | M | L | 0/4 |
| 119 | | 82 | 20 | W | L | 0/11 |
| 133 | | 43 | 16 | M | L | 0/8 |
| 022 | | 49 | 40 | W | L | 1/7 |
| 065 | | 69 | 18 | M | L | 1/9 |
| 066 | | 74 | 17 | W | L | 1/9 |
| 093 | | 72 | 20 | P | L | 2/9 |
| *DNA-aneuploid and intermediate/high-proliferative invasive carcinomas* | | | | | | |
| 123 | | 41 | 24 | P | H | 0/12 |
| 126 | | 84 | 18 | P | H | 0/7 |
| 135 | | 36 | 25 | P | H | 0/8 |
| 053[c] | | 86 | 47 | P | IM | [d] |
| 060 | | 78 | 70 | P | H | 7/7 |
| 080 | | 73 | 15 | P | H | 1/4 |
| 083 | | 81 | 20 | P | H | 4/6 |
| 116 | | 78 | 110 | P | IM | 8/10 |

[a]Histopathological differentiation: P, poor; M, moderate; W, well. [b]Proliferation (MIB 1): L, low (0–20% positive tumour cells); IM, intermediate (20–50%); H, high (>50%). [c]Mixed type, mucinous. [d]Distant metastases in the skeleton found. ND = not determined.

cells.Tumours with a single stem line in the diploid region were classified as diploid, and tumours with pronounced scattered DNA values exceeding the tetraploid region were classified as aneuploid (Auer *et al.*, 1980). Normal lymphocytes were used as internal 2c reference cells. Tumours with unclear classification were excluded from the study (12 cases).

### *Electrophoresis*

2-DE was performed by standard procedures as described previously (Franzén *et al.*, 1993; Anderson *et al.*, 1992). Resolyte (2%, pH 4–8; BDH) was used for isoelectric focusing, and 10–13% linear gradient sodium dodecyl sulphate (SDS) polyacrylamide gels in the second dimension. Gels were stained with silver nitrate according to standard procedures.

### *Identification of polypeptides*

A number of polypeptide spots were identified by matching with published maps and/or exchanging samples with other investigators. A rat embryonal fibroblast cell line WT2 (a kind gift from Drs JI Garrels and S Pattersson, Cold Spring Harbor, NY, USA) was used for the identification of a number of heat shock and structural proteins. 2-DE maps were prepared from WT2 cells and matched with the REF52 database (Garrels *et al.*, 1989). 2-DE maps were prepared from pre-B-ALL cells (clinical sample of acute lymphatic leukaemia) and subsequently analysed by Dr SM Hanash (University of Michigan, Ann Arbor, MI, USA). In addition, 2-DE maps were prepared from MRC-5 cells (cell lysate provided by JE Celis) and analysed by JE Celis (Aarhus University, Denmark).

The identity of some polypeptides was confirmed by purification–enrichment. Tropomyosins were purified as described by Matsumura *et al.* (1985) from W138 fibroblasts. The identification of GT($\pi$) was confirmed by an *in vitro* drug resistance experiment, where vincristine-resistant human K562 cells (cell pellets provided by S Vitols, Karolinska Institutet, Stockholm) were found to overexpress the polypeptide which had tentatively been identified as GT($\pi$). Cytokeratins were extracted from MCF-7 cell lysates (Paulin *et al.*, 1980). Proliferating cell nuclear antigen (PCNA) was identified by immunoblotting (PC10 MAb, Dakopatt) using a semi-dry system (Multiphor II Nova Blot, Pharmacia Biotech AB) and electrochemiluminescence detection (Amersham).

### *Scanning and image analysis*

2-DE gels were scanned at 100 $\mu$m resolution using Molecular Dynamics laser densitometer. Data were analysed using the PDQUEST software (Garrels *et al.*, 1984) (Pharmacia Biotech, Uppsala, Sweden). A synthetic 'identification reference pattern' including at least all identified spots was constructed. In subsequent analyses, polypeptide spots were matched to spots in the reference pattern using the PDQUEST software (construction of a 'matchset'). Background was subtracted, peaks located and the individual polypeptide quantities were expressed as p.p.m. of the total integrated optical density. Three groups were constructed within the matchset: benign lesions, DNA-diploid/low-proliferative tumours and DNA-aneuploid/high-proliferative tumours. The level of polypeptide expression in each group was calculated as the mean value ($\pm$standard deviation) of normalised p.p.m. values. We used the Mann–Whitney non-parametric test for determination of significant differences at the levels of $P<0.05$.

### Results

### *Analysis of polypeptide expression in breast cancer lesions*

A total of 23 lesions were examined. Of these, six cases were non-malignant (four fibroadenomas) and 17 cases were

**Figure 1** Reference 2-DE gel profile assembled from breast carcinomas and MDA-MB-231 breast cells. Acidic polypeptides are to the left. Polypeptides with known identity are encircled. Polypeptides with names within brackets were tentatively identified.

invasive, ductal breast carcinomas (Table I). Carcinomas were divided into two groups: (1) slowly proliferating and diploid tumours and (2) rapidly proliferating and aneuploid tumours as described in Materials and methods. The histopathological characteristics of all cases are presented in Table I. Cases not conforming to this classification were not considered.

Cells were extracted from fresh tumour tissue and single-cell suspensions free of erythrocytes were prepared as previously described (Franzén *et al.*, 1993; see Materials and methods). These preparations were usually >90% tumour cells. Samples were prepared for two-dimensional gel electrophoresis (2-DE). The polypeptide patterns were analysed by PDQUEST software (Garrels *et al.*, 1984), and the levels of individual polypeptides were expressed as p.p.m. The identity of individual spots was ascertained by co-electrophoresis of purified polypeptides and/or matching with databases (see Material and methods). Shown in Figure 1 is a 'reference pattern' assembled from tumours and one breast cancer cell line (MDA-MB-231).

*Evaluation of 2-DE gels from non-malignant and malignant breast lesions*

Figure 2 shows representative examples of gels derived from a fibroadenoma, a slowly proliferating carcinoma and a rapidly proliferating carcinoma. A 'window' of neutral to acidic proteins in the 25–100 kDa range is shown. Differences in polypeptide expression will be discussed below for various polypeptides of which the identities are known (encircled in Figures 1 and 2). Mean values of spot intensities in different groups of lesions are presented in Table II.

*Cell cycle-related proteins*

The recorded spot intensity of PCNA (proliferating cell nuclear antigen) was found to reflect the proliferative status of the tumours. PCNA levels were 4.3-fold higher (significant, see Materials and methods) in highly malignant tumours than in non-malignant cells (Table II). The distribution of PCNA levels

**Figure 2** 2-DE profiles from (**a**) a case of fibroadenoma, (**b**) a case of slowly proliferating carcinoma and (**c**) a case of rapidly proliferating carcinoma.

**Table II** Polypeptide expression in breast lesions. The levels of individual polypeptides are expressed as p.p.m. of total polypeptides

| | *Levels* | | | | |
|---|---|---|---|---|---|
| *Polypeptide* | *Benign lesions* *Mean*±s.d. (n=*6*) | *Diploid/low proliferative lesions* *Mean*±s.d. | (n=*9*) | *Aneuploid/high proliferative lesions* *Mean*±s.d. | (n=*8*) |
| PCNA | 90±59 | 328±218 | (1.73)[a] | 821±341 | (4.32)[a] |
| Op18 | 77 | 21 | (0.27) | 238 | (3.09) |
| Tropomyosin 1 (TM1) | 747±285 | 172±152 | (0.23) | 503±395 | (0.71) |
| Tropomyosin 2 (TM2) | 485±135 | 50 | (0.10) | 149 | (0.30) |
| Tropomyosin 3 (TM3) | 1419±385 | 476±251 | (0.33) | 638±449 | (0.45) |
| Cytokeratin 8 (CK8) | 6158±4088 | 1895±1142 | (0.31) | 1107±1004 | (0.18) |
| Cytokeratin 18 (CK18) | 3685±1603 | 681±682 | (0.18) | 751±794 | (0.20) |
| Heat shock protein 27 (hsp 27) | 1687±828 | 954±386 | (0.56) | 2002±1042 | (1.19) |
| Heat shock protein 60 (hsp 60) | 4120±1111 | 5068±1498 | (1.23) | 5574±1521 | (1.35) |
| Hsp 60, phosphorylated (phsp 60) | 1669±750 | 2401±958 | (1.44) | 3618±1085 | (2.17) |
| Heat shock protein 73 (hsp 73) | 3422±936 | 4473±1998 | (1.31) | 4964±3854 | (1.45) |
| Heat shock protein 90 (hsp 90) | 658±423 | 1198±1211 | (1.82) | 1303±770 | (1.98) |
| Calreticulin (CALR) | 7104±2356 | 7569±3080 | (1.07) | 11961±7143 | (1.68) |
| Mitcon 3 | 3462±855 | 3769±1830 | (1.09) | 3313±2196 | (0.96) |
| eIF 5A | 1027±596 | 958±1637 | (0.93) | 946±785 | (0.92) |
| EF 1β | 2325±609 | 2265±801 | (0.97) | 1604±720 | (0.69) |
| LDH | 1622±639 | 1881±915 | (1.16) | 1661±710 | (1.02) |
| Annexin V | 4005±1013 | 3326±1767 | (0.83) | 5051±3566 | (1.26) |
| nm 23 | 711±359 | 1008±416 | (1.42) | 814±391 | (1.14) |
| SOD | 1316±857 | 2074±999 | (1.58) | 1777±1031 | (1.35) |
| GT(π) | 2381±1106 | 1679±2191 | (0.70) | 1460±1127 | (0.61) |

[a]Fold change relative to the mean value level of benign lesions.

in various samples is shown in Figure 3. Op18 (oncoprotein 18/stathmin) is a phosphoprotein believed to have a regulatory role in the cell cycle. Op18 could not be detected in eight out of nine slowly proliferating/diploid tumours, but was detected in five out of eight rapidly proliferating/aneuploid tumours (Figure 3). Higher Op18 levels were found in non-malignant lesions than in slowly proliferating carcinomas (Table II).

### *Cytoskeletal proteins*

A number of cytoskeletal proteins were identified in the polypeptide maps. These include actin, tropomyosin 1–5, α-actinin, α- and β- tubulin and cytokeratins 7, 8, 15 and 18. Some of these proteins were not well resolved from neighbouring spots, others could not be quantified because of overstaining. The identity of cytokeratin 8 and 18 could be confirmed by extracting cytoskeletal polypeptides from MCF-7 breast carcinoma cells (Figure 4). Cytokeratins 7 and 15 were tentatively identified by their (1) migration properties (spot position), (2) absence in fibroblasts, and (3) enrichment in cytoskeletal fractions from tumours. Tumour cytoskeleton preparations were, however, contaminated by cytosolic proteins, and the identification of cytokeratins 7 and 15 is considered as tentative. Cytokeratin 19 was identified using the MCF-7 extract but was not included in the analysis because of possible comigration with a large number of neighbouring spots in most of the cases.

All four forms of cytokeratins resolved were found to be expressed at lower levels (significant) in carcinomas than in fibroadenomas (Table II). The levels of cytokeratin 8 and 18 in individual tumours is shown in Figure 4. In 12 out of 17 carcinomas, cytokeratin 18 could not be detected due to low expression.

Tropomyosin levels were lower in carcinomas than in non-malignant cells, confirming our previous findings (Franzén *et al*., 1996). In the material analysed here, the decreases in tropomyosin 2 and 3 were statistically significant, but not that of tropomyosin 1.

**Figure 3** Levels of polypeptides in individual tumours. Shown are the relative levels of cytokeratin 8 (CK8), cytokeratin 18 (CK18), proliferating cell nuclear antigen (PCNA), oncoprotein 18 (Op18), tropomyosin 2 (TM2), tropomyosin 3 (TM3), heat shock protein 90 (HSP90) and phosphorylated heat shock protein 60 (pHSP60).

*Stress proteins*

Overexpression of some stress proteins, such as HSP90 and HSP27, have been associated with malignancy. We noticed a moderate increase in HSP90 (1.6-fold) and calreticulin (1.7-fold) in highly malignant cancers. HSP27 levels did not, in contrast, differ between carcinomas and non-malignant cells.

Whereas the levels of HSP60 showed small variations (1.3-fold higher in rapidly proliferating carcinomas), the levels of the phosphorylated form of this gene product, pHSP60, were 2.2-fold increased in rapidly proliferating carcinomas. This increase was statistically significant.

*nm23*

nm23 (Nucleotide diphosphate kinase) levels have been reported to be low in metastatic cells (Steeg *et al.*, 1993). A weak, but not significant, increase in nm23 levels was preferentially recorded in slow proliferating carcinomas. In the material analysed here, we could not find any association with lymph node status.

*Glutathione S-transferase π*

GT-π has been implicated in tumour progression and in resistance to chemotherapy (Daniel *et al.*, 1993). In the material studied here, GT-π levels were somewhat lower in malignant cells than in fibroadenomas. This difference was not statistically significant.

*Constitutively expressed proteins*

The levels of a number of identified proteins did not differ (less than 50% change) between groups. Mitcon3 (mitochondrial), eIF5A (initiation factor 5A), LDH (lactate dehydrogenase form M), annexin V and EF1b (elongation factor 1β) belonged to this category.

### Discussion

Analysis of polypeptide profiles in human tumours is not a trivial task. Samples may contain relatively large amounts of protein from other cell types present in the tumours, such as stromal fibroblasts and lymphocytes. Furthermore, massive contamination by serum proteins may preclude large areas of the maps. These problems can be circumvented by purifying viable tumour cells from freshly excised tumours (Franzén *et al.*, 1993). We have studied here the expression of 24 polypeptides with known identity (by name) in breast carcinomas and show that changes in the levels of some of these polypeptides can be demonstrated. As more than 1000 polypeptides have been identified in the human 2-DE map (Celis *et al.*, 1995), it will be possible to make detailed characterisations of changes in gene expression in tumours in the future.

**Figure 4** Purification of cytokeratins from MCF-7 breast carcinoma cells. 2-DE gels showing (A) total proteins and (B) extracted intermediate filaments are shown.

Tumours were classified as slowly proliferating/diploid or rapidly proliferating/aneuploid. Using this classification, we were convinced that the procedures used were sufficiently accurate to detect changes in the levels of proliferation markers. The levels of the PCNA polypeptide were found to increase in parallel with progression from non-malignant lesions to slowly proliferating and then to rapidly proliferating carcinomas. Similarly, oncoprotein 18 levels were 3-fold higher in rapidly growing tumours than in fibroadenomas.

Trask *et al.* (1990) reported that normal cultured breast epithelial cells produce cytokeratin 5, 6, 7, 14 and 15, whereas tumour cells produce mainly cytokeratin 8, 18 and

19. We found cytokeratins 7, 8, 15 and 18 to be expressed in fibroadenomas. This result suggests that alterations in cytokeratin expression occur early during neoplastic transformation and is consistent with the finding of low levels of cytokeratin 5 and high levels of cytokeratin 18 in immortalised breast cell lines (Trask *et al.*, 1990). The levels of cytokeratin 7, 8, 15 and 18 were significantly lower in carcinomas than in fibroadenomas. Decreases in cytokeratin immunostaining in breast cancer have been previously described (Wada *et al.*, 1991; Takei *et al.*, 1995; Heatley *et al.*, 1995). Takei *et al.* (1995) reported that cytokeratin 8 (CK8) staining was negative in 35% of invasive breast carcinomas examined, and that the absence of CK8 correlated with oestrogen receptor negativity. Similarly, Heatley *et al.* (1995) reported that some carcinomas are negative for cytokeratins 7, 8 or 18. Paine *et al.* (1992) showed that *in vitro* transformation of MCF-10A cells with *ras*-oncogenes decreased the levels of cytokeratins 7, 8, 15 and 16. Our data extend previous reports, as we can demonstrate decreases in cytokeratins in *ex vivo* tumour cells by measurements of polypeptide levels. We can exclude the possibility that previously reported decreases in immunostaining could be explained by the masking of epitopes or as the result of immunohistochemical artifacts.

Cytokeratins are used as markers to identify breast carcinoma cells in various situations (for a review, see Moll *et al.*, 1991). In two colour multiparametric flow cytometry analyses, the epithelial cell compartment is identified by cytokeratin antibodies, and DNA histograms can be specifically obtained from these cells (Ramaekers *et al.*, 1984; Wingren *et al.*, 1994). MAbs to cytokeratins have been used to detect epithelial tumour cells that have metastasised from primary adenocarcinomas to secondary sites such as the bone marrow (Pantel *et al.*, 1993; Harbeck *et al.*, 1994). Pantel *et al.* (1993) reported that the incidence of metastatic cells in bone marrow was 74% in breast cancer patients known to have macroscopical metastases. Decrease or loss of cytokeratins in carcinomas can potentially be a problem in these types of studies, as the most malignant cells may escape detection.

Deregulation of tropomyosin expression has been shown to contribute to morphological transformation in experimental systems. It was recently suggested that Fos oncoproteins induce deregulation of genes encoding cytoskeleton-associated proteins (Jooss *et al.*, 1995). We have previously observed down-regulation of tropomyosin 1–3 in breast carcinoma tissue (Franzén *et al.*, 1996). In the present study, significant down-regulation was observed for tropomyosin 2 and 3. In one of the tumours, high tropomyosin expression was observed (see Figure 3). Whether tropomyosin in these cells was due to contaminating, non-malignant cells or due to expression in tumour cells is not clear.

Increases in various stress proteins were found in carcinomas, but these were less than 2-fold. HSP-27 has been reported to be overexpressed in 25% of invasive ductal carcinomas, and overexpression in early-stage breast cancer is associated with poor prognosis. In addition to being induced by heat shock, HSP90 may be induced by transformation by *ras* (Lebeau *et al.*, 1991). HSP90 may also regulate DNA-binding activities of progesterone receptors in breast cancer cells. In previous studies, we observed elevated levels of HSP90 in potentially highly malignant breast tumours and in small-cell lung carcinomas (Okuzawa *et al.*, 1994) using 2-DE.

We conclude from this study that 2-DE can be used to study complex changes in gene expression occurring in tumours. We believe that with the advent of standardised 2-DE techniques and 2-DE data bases, this approach may be a useful complement to other techniques, such as cDNA library screening.

**Acknowledgements**
We are grateful to Ann Ohlsson, Kicki Svanholm, Birgitta Sundelin and Inga Maurin for technical assistance and Martin Bäckdal, Göran Wallin and Eva Edholm for tumour material. We also thank Sixten Franzén for valuable help with cytological evaluations. This work was supported by Cancerföreningen i Stockholm and by Cancerfonden.

### References

ANDERSON NL. (1992). *Two Dimensional Electrophoresis, Operation of the ISO-DALT System.* Large Scale Biology Press: Washington DC.

AUER GU, CASPERSSON T AND WALLGREN A. (1980). DNA content and survival in mammary carcinoma. *Anal. Quant. Cytol. Histol.*, **3**, 161–165.

CELIS JE, HOLM RASMUSSEN H, GROMOV P, OLSEN E, MADSEN P, LEFFERS H, HONORE B, DEJGAARD K, VORUM H, BACK KRISTENSEN D, ØSTERGARD M, HANSØ A, AAGARD JENSEN N, CELIS A, BASSE B, LAURIDSEN JB, RATZ GP, ANDERSEN AH, WALBUM E, KJAERGAARD I, ANDERSEN I, PUYPE M, VAN DAMME J AND VANDERKERCKHOVE J. (1995). The human keratinocyte two-dimensional gel protein database (update 1995): mapping components of signal transduction pathways. *Electrophoresis*, **16**, 2177–2240.

DANIEL V. (1993). Glutathione S-transferases: gene structure and regulation of expression. *Crit. Rev. Biochem. Mol. Biol.*, **28**: 173–207.

FRANZÉN B, OKUZAWA K, LINDER S, KATO H AND AUER G. (1993). Non-enzymatic extraction of cells from clinical tumour material for analysis of gene expression by two-dimensional gel electrophoresis. *Electrophoresis*, **14**, 382–390.

FRANZÉN B, LINDER S, URYU K, ALAIYA AA, HIRANO T, KATO K AND AUER G. (1996). Expression of tropomyosin isoforms in benign and malignant human breast lesions. *Br. J. Cancer*, **73**, 909–913.

GARRELS JI. (1979). Two-dimensional gel electrophoresis and computer analysis of proteins synthesized by clonal cell lines. *J. Biol. Chem.*, **254**, 7961–7977.

GARRELS JI, FARRAR JT AND BURWELL CB. (1984). The QUEST system for computer-analyzed two-dimensional gel electrophoresis of proteins. In *Two-Dimensional Gel Electrophoresis of Proteins: Methods and Applications*, Celis JE and Bravo R. (eds) pp. 37–91. Academic Press: New York.

GARRELS JL AND FRANZA BJ. (1989). Transformation-sensitive and growth-related changes of protein synthesis in REF52 cells. A two-dimensional gel analysis of SV40-, adenovirus-, and Kirsten murine sarcoma virus-transformed rat cells using the REF52 protein database. *J. Biol. Chem.*, **264**, 5299–5312.

HARBECK N, UNTCH M, PACHE L AND EIERMANN W. (1994). Tumour cell detection in the bone marrow of breast cancer patients at primary therapy: results of a 3-year median follow-up. *Br. J. Cancer*, **69**, 566–571.

HEATLEY M, MAXWELL P, WHITESIDE C AND TONER P. (1995). Cytokeratin intermediate filament expression in benign and malignant breast disease. *J. Clin. Pathol.*, **48**, 26–32.

JOOSS KU AND MULLER R. (1995). Deregulation of genes encoding microfilament-associated proteins during Fos-induced morphological transformation. *Oncogene*, **10**, 603–608.

LEBEAU J, LE CHALONY C, PROSPERI MT AND GOUBIN G. (1991). Constitutive overexpression of a 89 kDa heat shock protein gene in the HBL100 human mammary cell line converted to a tumorigenic phenotype by the EJ/T24 Harvey-ras oncogene. *Oncogene*, **6**, 1125–1132.

LINDER S, BRESKI H AND RINGERTZ NR. (1979). Phenotypic expression in cybrids derived from teratocarcinoma cells fused with myoblast cytoplasms. *Exp. Cell Res.*, **120**, 1–14.

MATSUMURA F AND YAMASHIRO-MATSUMURA S. (1985). Purification and characterization of multiple isoforms of tropomyosin from rat cultured cells. *J Biol. Chem.*, **260**, 13851–13859.

MOLL R. (1992). Molecular diversity of cytokeratins: significance for cell and tumor differentiation. *Acta Histochem.*, **41** (Suppl.), 117–127.

OKUZAWA K, FRANZÉN B, LINDHOLM J, LINDER S, HIRANO T, BERGMAN T, EBIHARA Y, KATO H AND AUER G. (1994). Characterization of gene expression in clinical lung cancer materials by two-dimensional polyacrylamide gel electrophoresis. *Electrophoresis*, **15**, 382–390.

PAINE TM, FONTANINI G, BASOLO F, GERONIMO I, ELLIOTT JW AND RUSSO J. (1992). Mutated c-Ha-ras oncogene alters cytokeratin expression in the human breast epithelial cell line MCF-10A. *Am. J. Pathol.*, **140**, 1483–1488.

PANTEL K, SCHLIMOK G, BRAUN S, KUTTER D, LINDEMANN F, SCHALLER G, FUNKE I, IZBICKI JR AND RIETHMULLER G. (1993). Differential expression of proliferation-associated molecules in individual micrometastatic carcinoma cells. *J. Natl Cancer Inst.*, **85**, 1419–1424.

PAULIN D, FOREST N AND PERREAU J. (1980). Cytoskeletal proteins used as marker of differentiation in mouse teratocarcinoma cells. *J. Mol. Biol.*, **144**, 95–101.

RAMAEKERS FC, BECK H, VOOIJS GP AND HERMAN CJ. (1984). Flow-cytometric analysis of mixed cell populations using intermediate filament antibodies. *Exp. Cell Res.*, **153**, 249–253.

STEEG PS, DE LA ROSA A, FLATOW U, MACDONALD NJ, BENEDICT M AND LEONE A. (1993). Nm23 and breast cancer metastasis (Review). *Breast Cancer Res. Treat.*, **25**, 175–187.

TAKEI H, IINO Y, HORIGUCHI J, KANOH T, TAKAO Y, OYAMA T AND MORISHITA Y. (1995). Immunohistochemical analysis of cytokeratin 8 as a prognostic factor in invasive breast carcinoma. *Anticancer Res.*, **15**, 1101–1105.

TRASK DK, BAND V, ZAJCHOWSKI DA, YASWEN P, SUH T AND SAGER R. (1990). Keratins as markers that distinguish normal and tumor-derived mammary epithelial cells. *Proc. Natl Acad. Sci. USA*, **87**, 2319–2323.

WADA T, YASUTOMI M, YAMADA K, HASHIMURA K, KUNIKATA M, TANAKA T, HUANG JW AND MORI M. (1991). Heterogeneity of keratin expression and actin distribution in benign and malignant mammary diseases. *Anticancer Res.*, **11**, 1983–1993.

WINGREN SOS AND NORDENSKJÖLD B. (1994). Flow cytometric analysis of S-phase fraction in breast carcinomas using gating on cells containing cytokeratin. South East Swede Breast Cancer Group. *Br. J. Cancer*, **69**, 546–549.


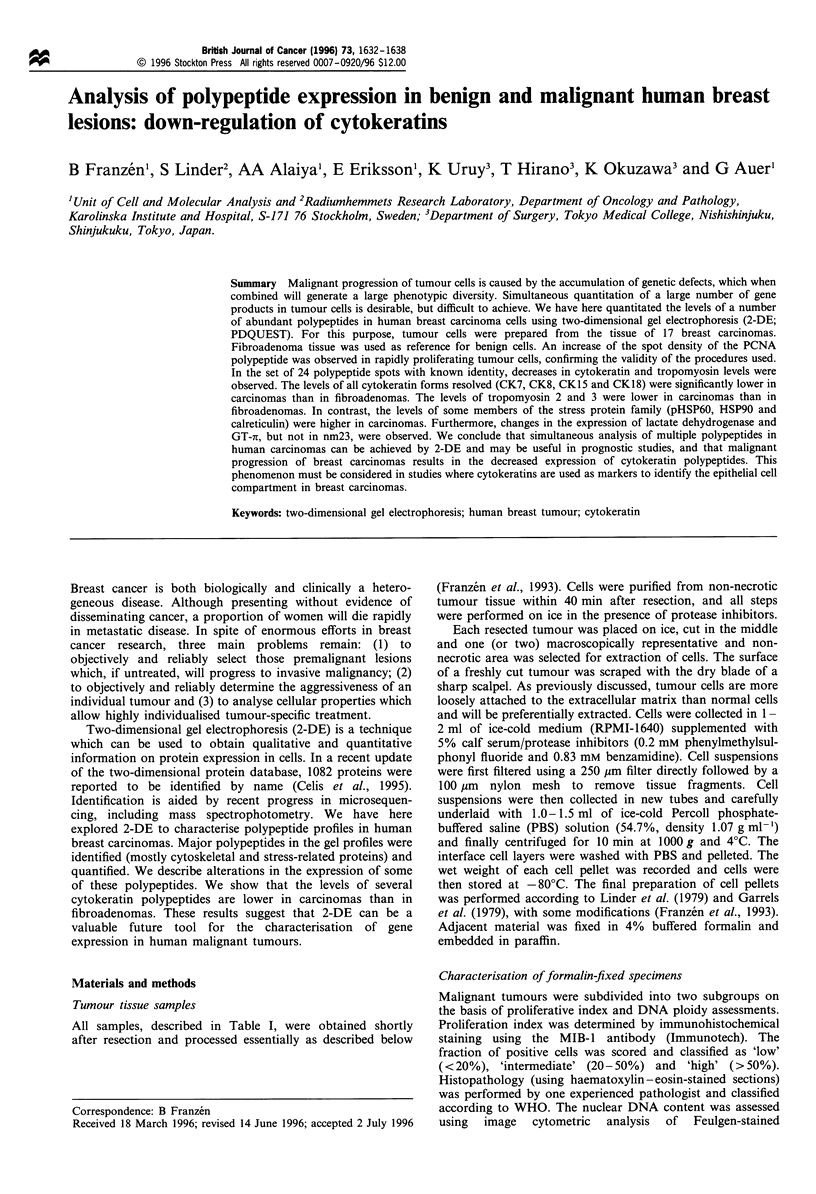

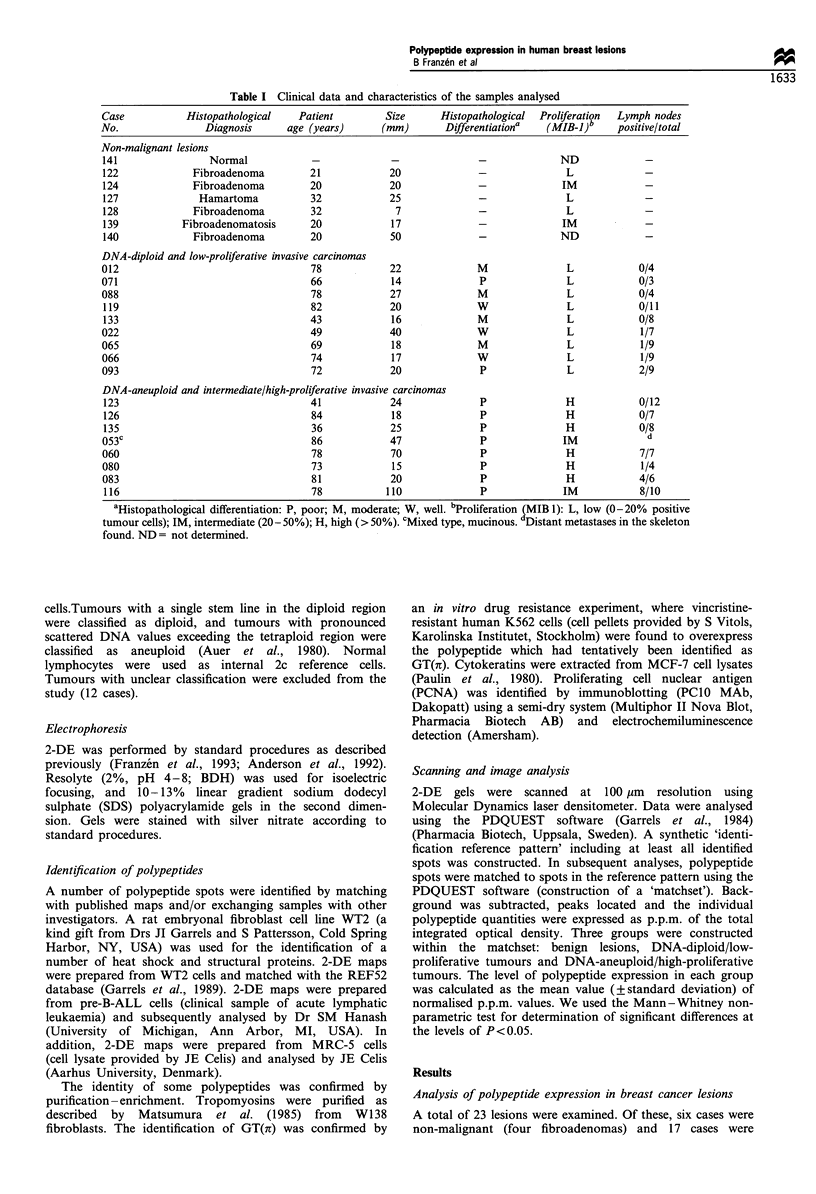

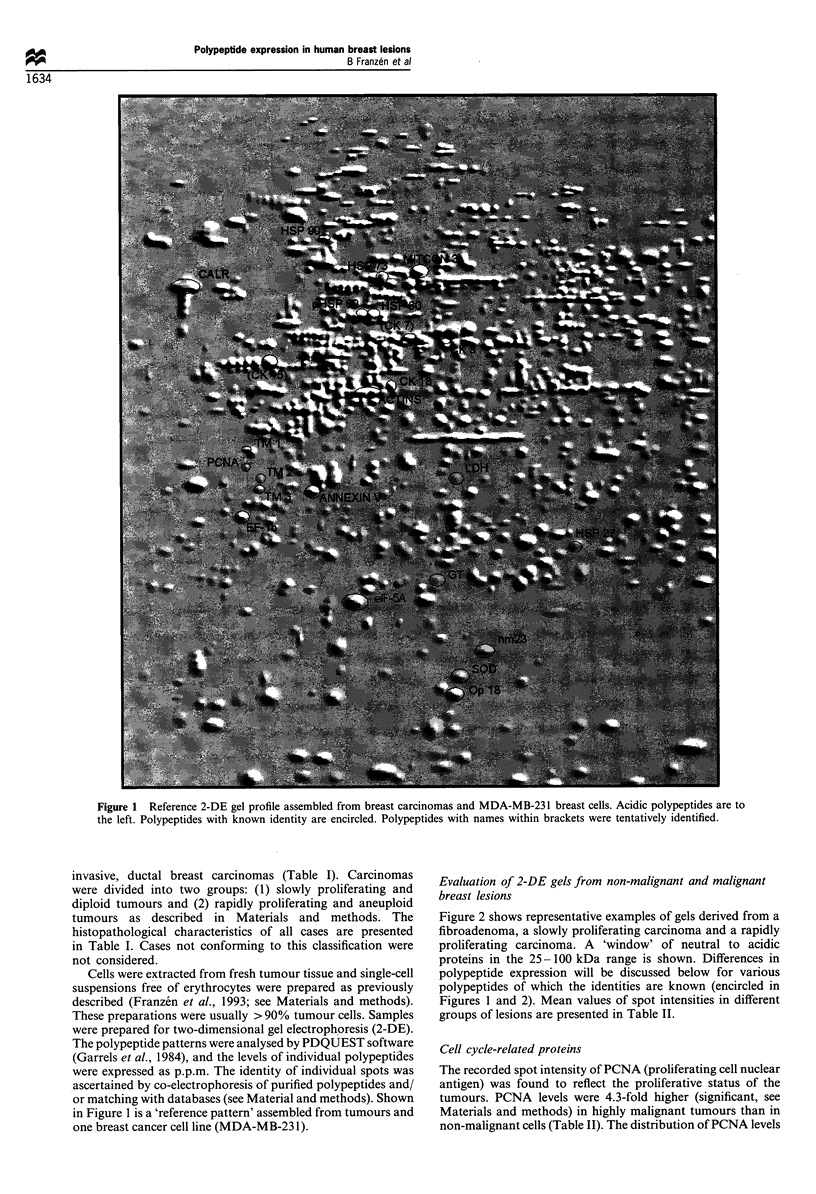

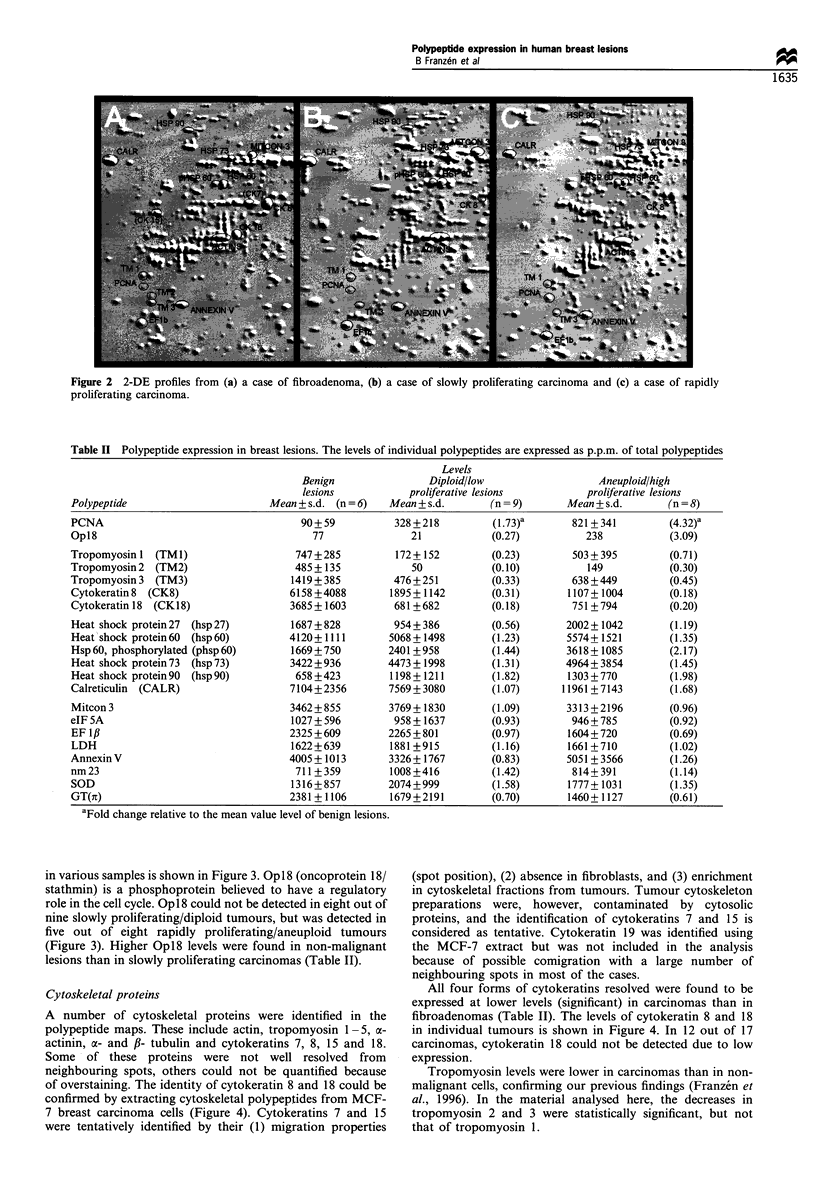

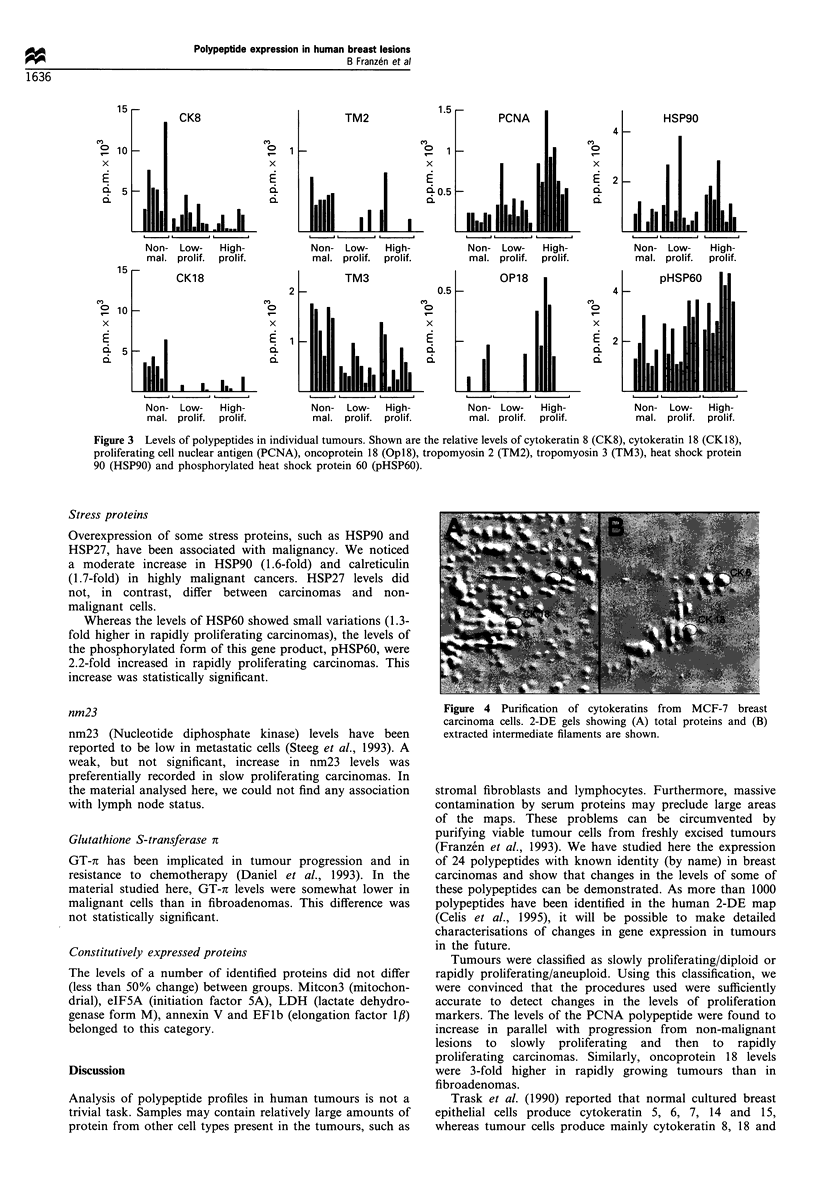

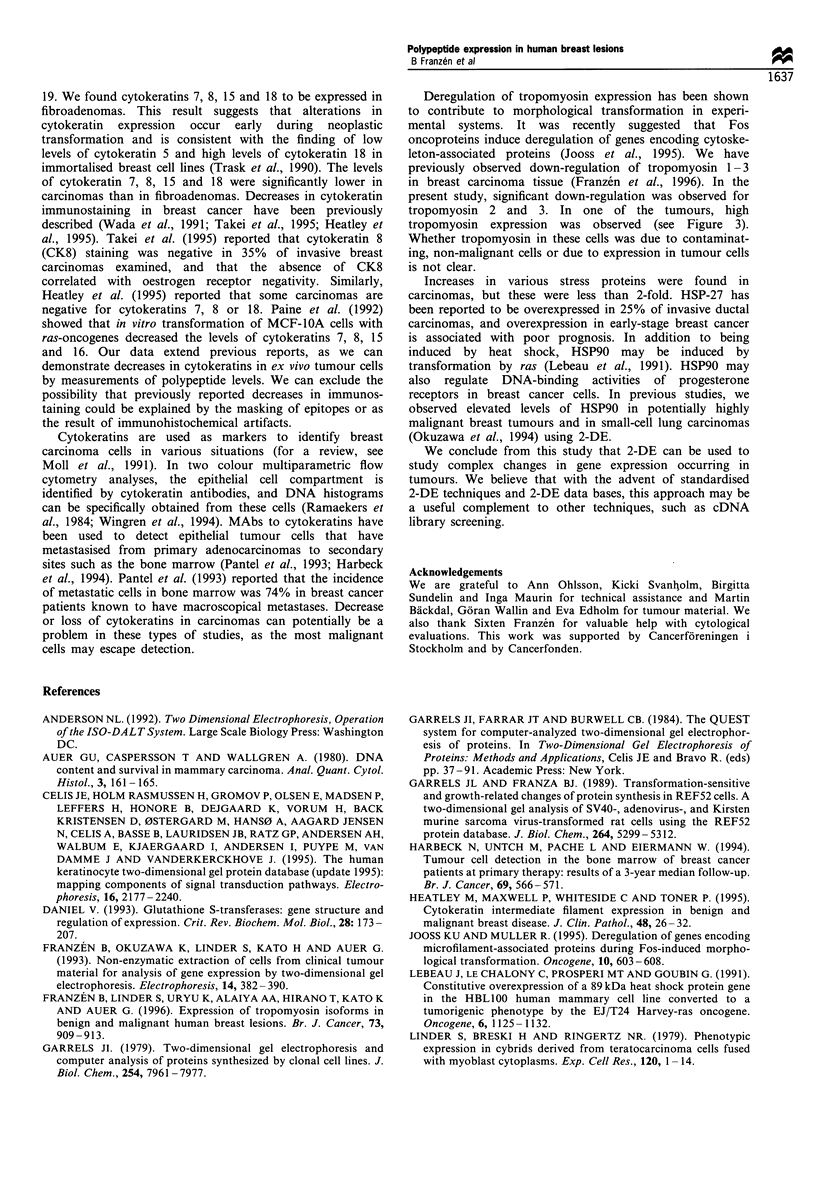

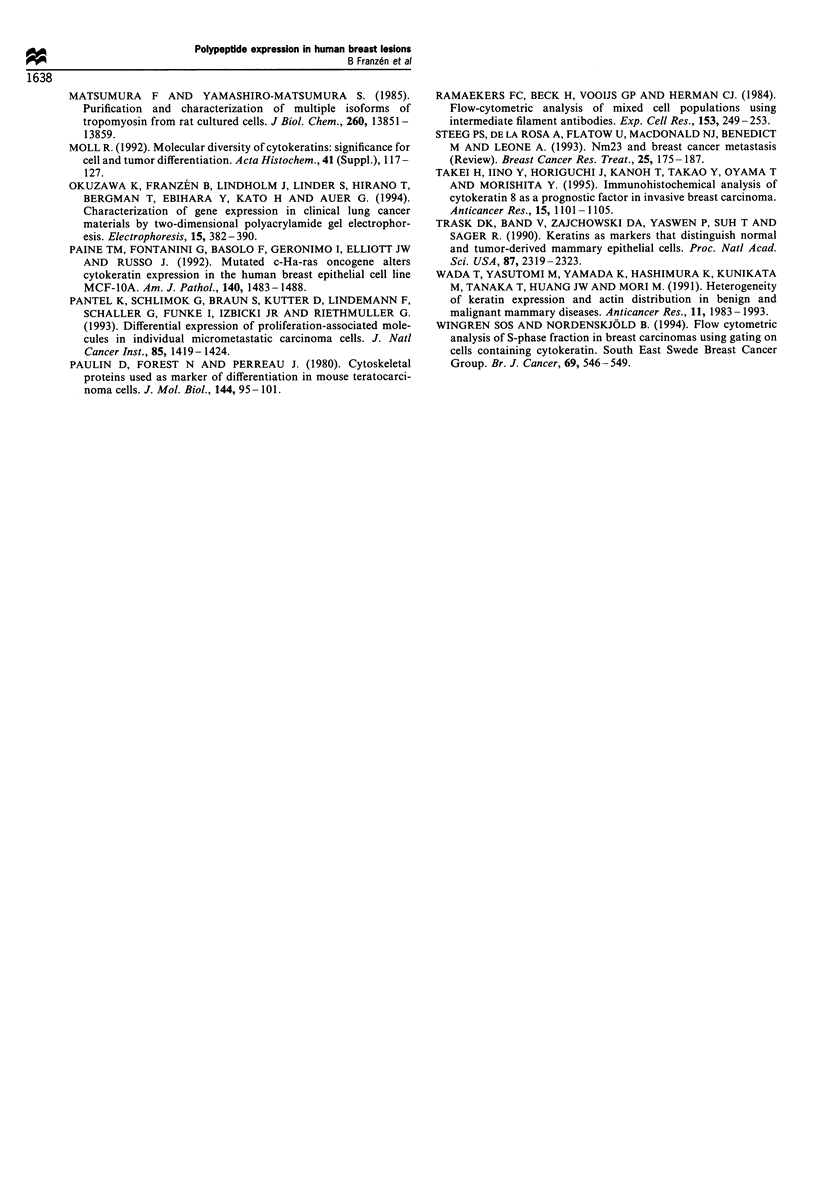


## References

[OCR_00809] Auer G. U., Caspersson T. O., Wallgren A. S. (1980). DNA content and survival in mammary carcinoma.. Anal Quant Cytol.

[OCR_00816] Celis J. E., Rasmussen H. H., Gromov P., Olsen E., Madsen P., Leffers H., Honoré B., Dejgaard K., Vorum H., Kristensen D. B. (1995). The human keratinocyte two-dimensional gel protein database (update 1995): mapping components of signal transduction pathways.. Electrophoresis.

[OCR_00827] Daniel V. (1993). Glutathione S-transferases: gene structure and regulation of expression.. Crit Rev Biochem Mol Biol.

[OCR_00838] Franzén B., Linder S., Uryu K., Alaiya A. A., Hirano T., Kato H., Auer G. (1996). Expression of tropomyosin isoforms in benign and malignant human breast lesions.. Br J Cancer.

[OCR_00856] Garrels J. I., Franza B. R. (1989). Transformation-sensitive and growth-related changes of protein synthesis in REF52 cells. A two-dimensional gel analysis of SV40-, adenovirus-, and Kirsten murine sarcoma virus-transformed rat cells using the REF52 protein database.. J Biol Chem.

[OCR_00844] Garrels J. I. (1979). Two dimensional gel electrophoresis and computer analysis of proteins synthesized by clonal cell lines.. J Biol Chem.

[OCR_00861] Harbeck N., Untch M., Pache L., Eiermann W. (1994). Tumour cell detection in the bone marrow of breast cancer patients at primary therapy: results of a 3-year median follow-up.. Br J Cancer.

[OCR_00869] Heatley M., Maxwell P., Whiteside C., Toner P. (1995). Cytokeratin intermediate filament expression in benign and malignant breast disease.. J Clin Pathol.

[OCR_00874] Jooss K. U., Müller R. (1995). Deregulation of genes encoding microfilament-associated proteins during Fos-induced morphological transformation.. Oncogene.

[OCR_00877] Lebeau J., Le Chalony C., Prosperi M. T., Goubin G. (1991). Constitutive overexpression of a 89 kDa heat shock protein gene in the HBL100 human mammary cell line converted to a tumorigenic phenotype by the EJ/T24 Harvey-ras oncogene.. Oncogene.

[OCR_00886] Linder S., Brzeski H., Ringertz N. R. (1979). Phenotypic expression in cybrids derived from teratocarcinoma cells fused with myoblast cytoplasms.. Exp Cell Res.

[OCR_00895] Matsumura F., Yamashiro-Matsumura S. (1985). Purification and characterization of multiple isoforms of tropomyosin from rat cultured cells.. J Biol Chem.

[OCR_00901] Moll R. (1991). Molecular diversity of cytokeratins: significance for cell and tumor differentiation.. Acta Histochem Suppl.

[OCR_00907] Okuzawa K., Franzén B., Lindholm J., Linder S., Hirano T., Bergman T., Ebihara Y., Kato H., Auer G. (1994). Characterization of gene expression in clinical lung cancer materials by two-dimensional polyacrylamide gel electrophoresis.. Electrophoresis.

[OCR_00913] Paine T. M., Fontanini G., Basolo F., Geronimo I., Elliott J. W., Russo J. (1992). Mutated c-Ha-ras oncogene alters cytokeratin expression in the human breast epithelial cell line MCF-10A.. Am J Pathol.

[OCR_00920] Pantel K., Schlimok G., Braun S., Kutter D., Lindemann F., Schaller G., Funke I., Izbicki J. R., Riethmüller G. (1993). Differential expression of proliferation-associated molecules in individual micrometastatic carcinoma cells.. J Natl Cancer Inst.

[OCR_00926] Paulin D., Forest N., Perreau J. (1980). Cytoskeletal proteins used as marker of differentiation in mouse terato;carcinoma cells.. J Mol Biol.

[OCR_00929] Ramaekers F. C., Beck H., Vooijs G. P., Herman C. J. (1984). Flow-cytometric analysis of mixed cell populations using intermediate filament antibodies.. Exp Cell Res.

[OCR_00937] Steeg P. S., de la Rosa A., Flatow U., MacDonald N. J., Benedict M., Leone A. (1993). Nm23 and breast cancer metastasis.. Breast Cancer Res Treat.

[OCR_00941] Takei H., Iino Y., Horiguchi J., Kanoh T., Takao Y., Oyama T., Morishita Y. (1995). Immunohistochemical analysis of cytokeratin #8 as a prognostic factor in invasive breast carcinoma.. Anticancer Res.

[OCR_00947] Trask D. K., Band V., Zajchowski D. A., Yaswen P., Suh T., Sager R. (1990). Keratins as markers that distinguish normal and tumor-derived mammary epithelial cells.. Proc Natl Acad Sci U S A.

[OCR_00951] Wada T., Yasutomi M., Yamada K., Hashimura K., Kunikata M., Tanaka T., Huang J. W., Mori M. (1991). Heterogeneity of keratin expression and actin distribution in benign and malignant mammary diseases.. Anticancer Res.

[OCR_00957] Wingren S., Stål O., Nordenskjöld B. (1994). Flow cytometric analysis of S-phase fraction in breast carcinomas using gating on cells containing cytokeratin. South East Sweden Breast Cancer Group.. Br J Cancer.

